# Stromal C-type lectin receptor COLEC12 integrates H. pylori, PGE2-EP2/4 axis and innate immunity in gastric diseases

**DOI:** 10.1038/s41598-018-20957-2

**Published:** 2018-02-28

**Authors:** Lin-Li Chang, Wen-Hung Hsu, Mou-Chieh Kao, Chih-Chung Chou, Chung-Cheng Lin, Chung-Jung Liu, Bi-Chuang Weng, Fu-Chen Kuo, Chao-Hung Kuo, Ming-Hong Lin, Chun-Jen Wang, Chun-Hung Lin, Deng-Chyang Wu, Shau-Ku Huang

**Affiliations:** 1 Department of Microbiology and Immunology, Faculty of Medicine, College of Medicine, Kaohsiung Medical University, Kaohsiung, Taiwan; 2 Graduate Institute of Medicine, College of Medicine, Kaohsiung Medical University, Kaohsiung, Taiwan; 3 Graduate Institute of Clinical Medicine, College of Medicine, Kaohsiung Medical University, Kaohsiung, Taiwan; 4 Center for Infectious Disease and Cancer Research, Kaohsiung Medical University, Kaohsiung, Taiwan; 5 Department of Medical Research, Kaohsiung Medical University Hospital, Kaohsiung, Taiwan; 6 Division of Gastroenterology, Department of Internal Medicine, Kaohsiung Medical University Hospital, Kaohsiung, Taiwan; 7 Institute of Molecular Medicine, National Tsing Hua University, Hsinchu, Taiwan; 8 Department of Life Science, National Tsing Hua University, Hsinchu, Taiwan; 9 School of Medicine, College of Medicine, E-Da Hospital, I-Shou University, Kaohsiung, Taiwan; 10 Center for Stem Cell Research, Kaohsiung Medical University, Kaohsiung, Taiwan; 11 Chemical Biology and Molecular Biophysics, Taiwan International Graduate Program and the Institute of Biological Chemistry, Academia Sinica, Taipei, Taiwan; 12 Institute of Biochemical Sciences, National Taiwan University, Taipei, Taiwan; 13 Department of Chemistry, National Taiwan University, Taipei, Taiwan; 14 Department of Internal Medicine, Kaohsiung Municipal Ta-Tung Hospital, Kaohsiung, Taiwan; 15 National Institute of Environmental Health Sciences, National Health Research Institutes, Miaoli, 35053 Taiwan; 16 Research Center for Environmental Medicine, Kaohsiung Medical University, Kaohsiung, 80708 Taiwan; 17 Shen-Zhen University Lo-Hu Hospital, Shen-Zhen, China; 18 Johns Hopkins Asthma and Allergy Center, School of Medicine, Johns Hopkins University, Baltimore, Maryland 21224 USA

## Abstract

Tissue stroma is known to be important in regulating Hp-mediated inflammation, but its interaction with Hp and dendritic cells (DCs) remains to be determined. To this end, the potential crosstalk between *H. pylori* (Hp) infected gastric stromal cells (Hp-GSCs) and DCs was investigated. Primary GSCs from cancerous and adjacent normal tissues were generated from gastric cancer patients, and monocyte-derived DCs were obtained from healthy individuals. Levels of cytokines and prostaglandin E_2_ (PGE_2_) were measured by ELISA, and C-type lectin expression in GSCs was assessed by flow cytometry and immunohistochemistry. In a trans-well co-culture system, significantly upregulated DC-derived IL-23 expression was found when DCs were co-cultured with Hp-infected GSCs (Hp-GSCs). Further, PGE_2_ from Hp-GSCs was discovered to possess the priming effect, which could be inhibited by anti-COLEC12 (Collectin subfamily member 12) Abs, COLEC12 knockdown or when alpha3-fucosyltransferase-null (*futB*; HP0651) strain of Hp was used. Also, the expression of COLEC12 was co-localized with CD90^+^ stromal cells in cancerous tissues. Hp-GSCs-conditioned DCs were able to induce the expression of IL-17 from CD4^+^ T cells, which could be inhibited by IL-23-neutralizing Abs. These results suggested the importance of COLEC12 as a receptor involved in Hp-stromal cell interaction and its subsequent conditioning effect on DCs.

## Introduction

Hp infection with chronic inflammation is still the high risk factor for gastric cancer^1^. Growing evidence indicates that in response to Hp infection^2^, dendritic cells (DCs), gastric epithelial cells, and gastric stromal cells (GSCs) are important in the regulatory network, representing, collectively, the first line of defense. However, the underlying mechanisms in promoting chronic inflammation and carcinogenesis are still unclear. Studies concerning the contribution of GSCs in gastric diseases have been scarce, and the mechanisms through which Hp influences GSCs and DC’s function remain to be elucidated.

Hp is known to be able to disrupt the epithelial barrier, leading to the transit of Hp to the sub-epithelial mucosal layer^3,4^, where the contact between Hp and stromal cells is plausible and is potentially critical in controlling Hp-mediated inflammation. Indeed, stromal cells are important players in the tumor microenvironment and play a crucial role in carcinogenesis and inflammation^5,6^. Moreover, it has been shown that stromal cells are able to induce Th17 during Hp infection^7^, and CD90^+^ stromal cells can, perhaps, act as antigen presenting cells in the colon^8^. It is known that inhibition of the activation of these stromal cells leads to a decrease in tumor size in colon cancer animal models^9^. Further, a recent study by Bimczok *et al*. has suggested that stromal factors in normal gastric mucosa are able to regulate DC activation and the subsequent T-cell response, which could contribute to the permissiveness of both gastric and intestinal mucosa to colonize microbes^10^.

DCs are present underneath the gastric epithelium and suggested to be able to interact with Hp directly^2^ or could be regulated by Hp-infected gastric epithelium or stromal cells. Extensive efforts have been made regarding DC’s recognition of Hp, in part, through their innate pattern-recognition Toll-like receptors (TLRs) and C-type-lectin receptors (CLRs) to generate innate immunity and influence adaptive response^11,12^. CLRs are known to be crucial in recognition of complex glycan structures on various pathogens through their C-type carbohydrate-recognition domains (CRDs) and influence the subsequent adaptive immune response^13,14^. Much less is known, however, regarding the molecular basis of Hp and tissue stromal interaction, and it is currently unknown as to whether like DCs, stromal cells also utilize members of the CLR in recognition of Hp.

Upon Hp stimulation, DCs can produce cytokines that are important in orienting an immune response toward tolerance or immunity. Hp infection is associated with a marked mucosal induction of T helper (Th) type 1 and Th17-type cytokines that are governed by interleukin (IL)-12 and IL-23, respectively^15^. Increasing evidence has revealed the importance of IL-23 in linking innate and adaptive immunity through its ability in regulating IL-17 expression and Th17 development^16^. IL-23 is increased significantly in the gastric biopsies of patients infected with Hp^17–20^ and has been associated with several inflammatory diseases such as rheumatoid arthritis^21^, inflammatory bowel disease (IBD)^22^ and Hp-associated gastritis^15^. The relevance of IL-17 to Hp-associated inflammation has been proven by using animal models^23^. The recruitment of Th17 early in infection has been reported to be associated with inflammation and promotion of tumor growth and poor outcome^24–26^. Studies have revealed that Th17 cells are increased in gastric cancer patients, and that Hp infection increases IL-17 expression in the gastric mucosa of humans and animals experimentally^27,28^. Further, regulatory immune cells, mostly regulatory Foxp3^+^CD4^+^CD25^+^ T cells (Treg cells), have been identified as a major regulatory component of the adaptive immune response and are involved in Hp-related inflammation and bacterial persistence^29^, through, in part, their ability to suppress Th17 and IL-17-driven response.

These current results suggest, therefore, that mucosal gastric resident cells, such as stromal cells, may be critical in controlling mucosal immunity by virtue of their strategic location and their established role in dictating the adaptive immune response. To this end, we have utilized a trans-well co-culture system to investigate the possible role of GSCs and their secreted factors in the “cross-talk” with, and conditioning of, DCs. The results showed that PGE_2_, a lipid mediator, from Hp-infected GSCs was responsible for the induction of IL-23 from DCs and the subsequent generation of Th17 response, wherein a member of the CLR family, COLEC12, in GSCs was critical in mediating the crosstalk between GSCs and DCs.

## Methods

### Human subjects and ethical statement

Primary gastric stromal cells (GSCs) were obtained by endoscopic biopsy from gastric cancerous (GSC T2, T6, T9 and T21) or their adjacent normal tissues (GSC N2, N6, N9 and N21) of 4 patients with malignant neoplasm of the stomach after receiving total gastrectomy. In addition, a line of GSCs (GSC N1) from one Hp-negative patient with gastritis was available and used in this study for reference. Paraffin-embedded gastric surgical tissue specimens from the control and gastric cancer patients described above were also obtained. These primary GSCs and gastric surgical tissue blocks were collected with IRB-approved protocols at the Kaohsiung Medical University (KMUH-IRB-20130114). Peripheral blood samples obtained from human subjects were collected with IRB-approved protocols at the Kaohsiung Medical University (KMUH-IRB-20130308). Serum samples were collected from patients with gastritis without intestinal metaplasia [IM (−), n = 40], gastritis plus intestinal metaplasia [IM (+), n = 34] or gastric cancer (n = 83) before any treatment. Ten healthy individuals were collected and used as control (KMUH-IRB-20120176). The institutional review board of Kaohsiung Medical University, Kaohsiung, Taiwan approved these studies. Written informed consent was documented from all study subjects and all experiments were performed in accordance with relevant guidelines and regulations.

### Primary GSC culture and Hp

Primary gastric stromal cells (GSCs) were cultured in keratinocyte medium (rEGF, 5 ng/mL; BPE, 50 ug/mL; 10% FBS NAC; 2 mmol/L; Asc 2 P; 0.2 mmol/L, Gibco–Invitrogen Corporation, Grand Island, NY, USA). The purity of GSCs was analyzed by staining for stromal cell (primary fibroblast) marker CD90 (BioLegend, San Diego, USA). Hp 26695 and mutant strain (HP0651) of Hp 26695 lacking α3 fucosyltransferase gene (*futB)* were identified by biochemical tests.

### Generation of monocyte-derived DCs and naive CD4^+^ T cell isolation

Peripheral blood samples obtained from human subjects were free of Hp infection as determined by using the carbon 14 urea breath test (C14 UBT). CD14^+^ cells purified by sorting using anti-CD14-labeled magnetic beads (MACS, Miltenyi, Germany) from peripheral blood of healthy donors were cultured in the presence of GM-CSF (50 ng/ml; PeproTech, Rocky Hill, NJ, USA) and IL-4 (10 ng/ml; PeproTech, Rocky Hill, NJ, USA) to generate monocyte-derived DCs. Autologous CD4^+^ T cells were isolated from the PBMCs using a negative selection kit (MACS Miltenyi Biotec, GmbH, Germany) and activated with a combination of anti-CD3 (5 ug/ml; eBioscience, San Diego, CA, USA) and anti-CD28 Abs (2 ug/ml; eBioscience, San Diego, CA, USA).

### Co-culture of GSCs with DCs and measurement of cytokines and PGE_2_

This study utilized a trans-well co-culture system with an insert (0.45 μm, Corning, Corning, USA) to investigate the possible role of secreted soluble factors from GSCs or Hp-GSCs in the crosstalk with DCs. In the trans-well co-cultures, 2 × 10^5^ GSCs cells/well were plated with or without Hp infection at MOI = 1:200 in the apical side of the trans-well cultures, while a total of 2 × 10^5^ DCs were cultured in basolateral side for 24 h. DCs cultured alone, treated directly with Hp (MOI = 1:200) or LPS (100 ng/ml; E. coli, Sigma, USA) were used for comparison. Supernatants from the basolateral side were collected for analyses of cytokines, including IL-23, IL-10, IL-6, and IL-12 by ELISA (R&D Inc., Minneapolis, MN, USA). Supernatants from the apical or the basolateral side were collected for analyses of PGE_2_ by ELISA and of GM-CSF by cytokine array (R&D Inc., Minneapolis, MN, USA, ARY005). To investigate the effect of PGE_2_ on DC function, a COX-1 inhibitor, SC 560 (100 nM; Calbiochem, USA), a nonselective COX-1 and 2 inhibitor, indomethacin (100 nM; Sigma-Aldrich, USA), EP and DP1 receptor antagonist, AH-6809 (10 uM; Cayman Chemical, Ann Arbor, Michigan, USA), or EP4 receptor antagonist, AH 23848 (10 uM; Cayman Chemical, Ann Arbor, Michigan, USA) was added to the basolateral side of the co-cultures.

To evaluate the possible role of COLEC12 in GSCs in regulating PGE_2_ and IL-23 expression from GSCs and DCs, respectively, COLEC12-blocking Abs (100 μg/mL, R&D, USA) were added to the Hp treated GSCs or to the apical side of the trans-well co-cultures. After 24 h, supernatants from the Hp treated GSCs were collected for analysis of PGE_2_, and supernatants from the basolateral side of the co-cultures were harvested and determined for the levels of IL-23 by ELISA. For COLEC12 gene knockdown experiments, negative control siRNAs panel sequences (including 5′-UGGUUUACAUGUCGACUAA, 5′-UGGUUUACAUGUUGUGUGA, 5′-UGGUUUACAUGUUUUCUGA, and 5′-UGGUUUACAUGUUUUCCUA), and siRNA sequences directed against COLEC12 mRNA were purchased from Dharmacon (Chicago, USA). Four COLEC12 siRNA (50 nM) pool used included 5′-CGUCAGUAACCGUGCGAUU, 5′-GGUUAUCAUUGGUCGUUGA, 5′-GCCAAGAAGGACACGGAUU, and 5′-AUGGAAACAUCUCGCCAAA. GSC T21, and the cells were transduced with COLEC12 siRNA pool or negative control panel siRNAs by TransIT-X2 Dynamic Delivery System (Mirus Bio LLC 545 Science Drive, Madison, WI) for 72 h, followed by stimulating the cells with H. pylori for 24 h. Supernatants were collected for PGE_2_ analyses by ELISA. The efficiency of COLEC12 knockdown by siRNA was evaluated for the level of COLEC12 mRNA expression by q-PCR in GSC T21. To evaluate the possible role of LPS fucosylation pattern of Hp in regulating IL-23 expression, Hp 26695 mutant strain *FutB*, which lacks α3 fucosyltransferase gene, or wild-type strain was added in the trans-well co-culture system. In addition, for clarifying whether the glycan structure decorated with fucose/galactose confer the recognition specificity of COLEC12 on GSCs, blocking reagents, fucose (25 mM, Sigma, USA), galactose (25 mM, Sigma, USA), and a calcium chelator, EDTA (10 mM, Sigma, USA), were added for 1 h, then GSCs were stimulated with Hp for 24 h, and the supernatant was collected for measurement of PGE_2_. In some cases, 2 × 10^5^ DCs in basolateral side of the trans-well co-cultures described above were collected and washed twice in RPMI medium, and then were co-cultured with CD4^+^ T cells at a 1:5 (DC/CD4^+^ T) ratio. Supernatants were collected at day 3 and day 5 for the measurement of T-cell derived cytokines, including IL-17, IFN-γ and IL-4, by ELISA (R&D Inc, Minneapolis, MN, USA). To investigate the effect of IL-23 on IL-17 induction, an IL-23-neutralizing mAbs (1 mg/mL, R&D Inc., Minneapolis, MN, USA) was added.

### Analysis of CLR expression by flow-cytometry, immunohistochemistry and immunofluorescence

For flow-cytometry, 1 × 10^5^/ml GSCs were incubated with mouse anti-COLEC12 (1:50, Abnova, Taipei, Taiwan) or mouse anti-OLR1 (5 ul, R&D Inc, Minneapolis, MN, USA) Abs for 30 minutes. Secondary Abs, phycoerythrin conjugated goat anti-mouse IgG Abs (1:100, R&D Inc, Minneapolis, MN, USA), were added for 30 minutes. Surface markers on GSCs were analyzed using a FACScan flow cytometer and CellQuest software (Becton Dickinson). For immunohistochemistry, 1 × 10^5^/ml GSCs cultured on slides were incubated with mouse anti-COLEC12 (1:100, Abnova, Taipei, Taiwan) or mouse anti-OLR1 Abs (1:100, R&D Inc, Minneapolis, MN, USA), overnight at 4 °C in humidified chambers. Slides were further incubated with a biotinylated secondary Ab for 30 min at room temperature. Antigen-antibody complexes were detected by the avidin-biotin-peroxidase method using 3,3′-diaminobenzidine as a chromogenic substrate (Dako, Glostrup, Den-mark). Finally, the slides were counterstained with hematoxylin. For immunofluorescence, primary GSCs cultured on slides were fixed with 95% ethanol, and the expression of COLEC12 was detected using rabbit anti-COLEC12 (1:100, Nouvs, Littleton, USA) Abs. For gastric surgical tissue blocks, 5 μm-thick sections were cut, deparaffinized, and rehydrated. The expression of COLEC12 on CD90^+^ GSCs was detected using rabbit anti-COLEC12 Abs (1:100, Nouvs, Littleton, USA), or mouse anti-CD90 Abs (1:25, Biolegend, USA), overnight at 4 °C. Secondary Abs, goat anti-mouse IgG Cy3 (1:200, Jackson ImmunoResearch, West Baltimore Pike, USA) or Alexa 488 goat anti-rabbit Abs (1:200, Jackson ImmunoResearch, West Baltimore Pike, USA), were added for 1 h at 4 °C. Finally, DAPI (0.5 ug/ml, SIGMA, Saint Louis, USA) was added to slides for 1 h for nucleus staining. The images were captured with an Olympus FluoView 1000 confocal laser-scanning microscope (Olympus, Tokyo, Japan). As negative controls, the use of primary Abs was omitted or substituted with a relevant isotype control.

### Statistical analysis

Results are presented as mean ± SE. Analytical statistics were performed using the SPSS (version 20) software package. Statistical significance was calculated by nonparametric Mann-Whitney U test and for pair-wise comparisons only. In some cases, ANOVA followed by Scheffe multiple post hoc test were used. Differences were considered statistically significant at *P* < 0.05.

## Results

### Hp-infected GSCs (Hp-GSCs) regulated IL-23 expression in DCs and IL-17 expression from CD4^+^ T cells

To investigate the potential crosstalk between GSCs, a likely cell type directly in contact with Hp, and DCs, a trans-well co-culture system was used. The expression of IL-23 from DCs and the subsequent generation of IL-17 response were evaluated as the experimental readouts to represent the activation of DCs and Th17 cells, respectively, as the levels of IL-23 and IL-17 have been shown to be upregulated upon Hp infection in several human and murine models^17–19,30,31^.

**Figure 1:**
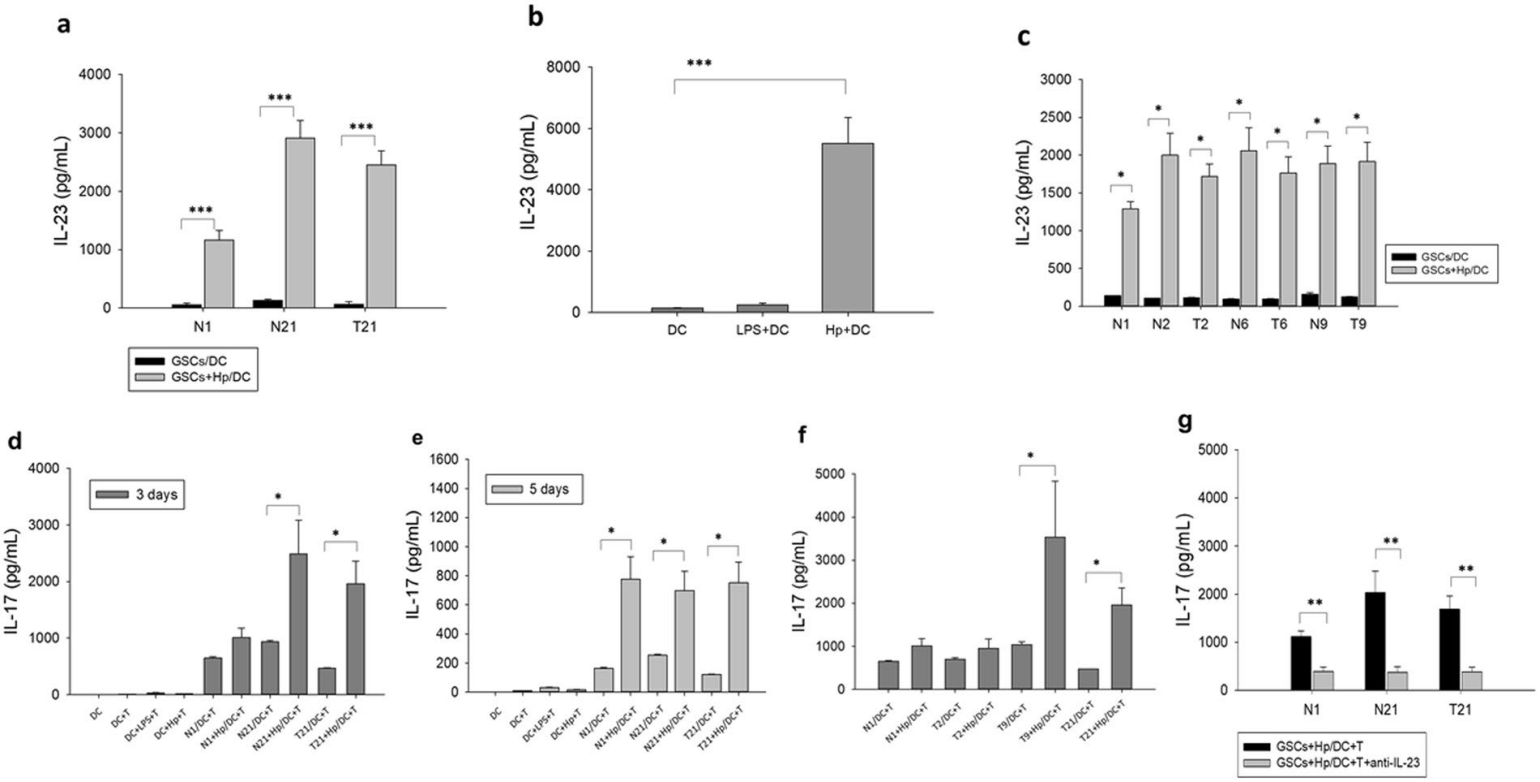
IL-23 secretion from DCs alone or in trans-well co-cultures with GSCs. (**a**) The level of IL-23 expression from DCs in trans-well co-cultures with GSCs of a non-cancer control individual (N1) and a cancer patient (T21, GSCs from cancer lesion; N21, GSCs from normal adjacent gastric tissue) in the absence or presence of Hp stimulation (Hp:MOI = 1:200) was analyzed by ELISA. Hp were added for 24 hrs in the upper chamber for stimulation of GSCs (2 × 10^5^ cells/ml). (**b**) The level of IL-23 expression from DCs in direct contact E. coli-derived LPS (100 ng/ml) or Hp for 24 hrs were used as control. (**c**) IL-23 expression in the other three sets of matched cancer GSCs (T2, T6, and T9) and adjacent normal GSCs (N2, N6, N9) obtained from gastric cancer patients. Hp-GSCs-conditioned DCs regulated IL-17 expression from CD4^+^ T cells. Level of IL-17 expression in CD4^+^ T cells co-cultured with conditioned DCs at a 1:5 (DC/CD4^+^ T) ratio was evaluated at day 3 (**d**) or day 5 (**e**). (**f**) IL-17 expression in three cancer GSCs (T2, T9, and T21) obtained from gastric cancer patients in the absence or presence of Hp stimulation (Hp:MOI = 1:200). (**g**) IL-17 production was measured in the presence or absence of IL-23-neutralizing Abs. The results are expressed as means ± SE, and the data shown represent independent experiments from three-five different DC donors. Mann- Whitney U test was used and for pair-wise comparisons only. *p < 0.05, **p < 0.01, ***p < 0.001.

We found that the level of IL-23 secretion from DCs was increased when DCs were co-cultured with Hp-primed GSCs (Fig. 1a). The enhancing effect on IL-23 expression was particularly noted when GSCs (T21) from the cancer lesion of a gastric cancer patient and its adjacent normal tissue (N21) was analyzed, while, as expected, stimulation of DCs alone with Hp induced appreciable levels of IL-23 (Fig. 1b). This finding suggested, therefore, that soluble factor(s) from Hp-infected GSCs mediated the increase of DC-derived IL-23 expression. Similarly, significantly increased levels of IL-23 were also found in the other three sets of Hp-primed primary cell GSCs (T2, T6, T9) and their adjacent normal pair tissue N2, N6, N9) from gastric cancer patients (Fig. 1c).

Next, to examine whether conditioned DCs as described above were able to enhance the Th17 response, conditioned DCs were then co-cultured with autologous CD4^+^ T cells for 3 and 5 days, and the supernatants in the basolateral side were collected for IL-17 measurement. Results showed that Hp-GSCs primed DCs increased IL-17 expression from CD4^+^ T cells at day 3 (Fig. 1d) and day 5 (Fig. 1e). Increased expression levels of IL-17 at day 3 were also found in the other Hp-primed gastric cancer GSCs T9 (Fig. 1f), which was significantly blocked by the addition of IL-23-neutralizing Abs (Fig. 1g). These findings suggested that soluble factor(s) from Hp-GSCs was able to induce IL-23 from DCs, which, in turn, generated Th17 response. The levels of IL-23 and IL-17 were also increased when GSCs from a non-cancer individual and gastric cancer patient were treated with Hp (Fig. 1a and d, respectively). It was noted also that co-incubation of DCs with Hp treated GSCs decreased the expression of maturation markers, HLA-DR and CD86 (Supplemental Fig. 1a and b, respectively) on DCs. In addition to IL-23, significantly increased levels of IL-10 (Supplemental Fig. 2a and b) and IL-6 (Supplemental Fig. 2c and d), but not IL-12 (Supplemental Fig. 2e and f), from DCs were found when co-cultured with Hp-GSCs. Further, when Hp-GSCs conditioned DCs were co-cultured with autologous CD4+ T cells for 3 days, decreased levels of IFN-γ, but not IL-4, were noted (Supplemental Fig. 2g and h, respectively). Collectively, these results suggested that Hp-primed GSCs were able to influence the innate DC’s response and the adaptive Th17 immunity, via likely the soluble factors from Hp-primed GSCs.

### PGE_2_ secreted from Hp-GSCs was found to be responsible for modulating DC-derived IL-23

**Figure 2:**
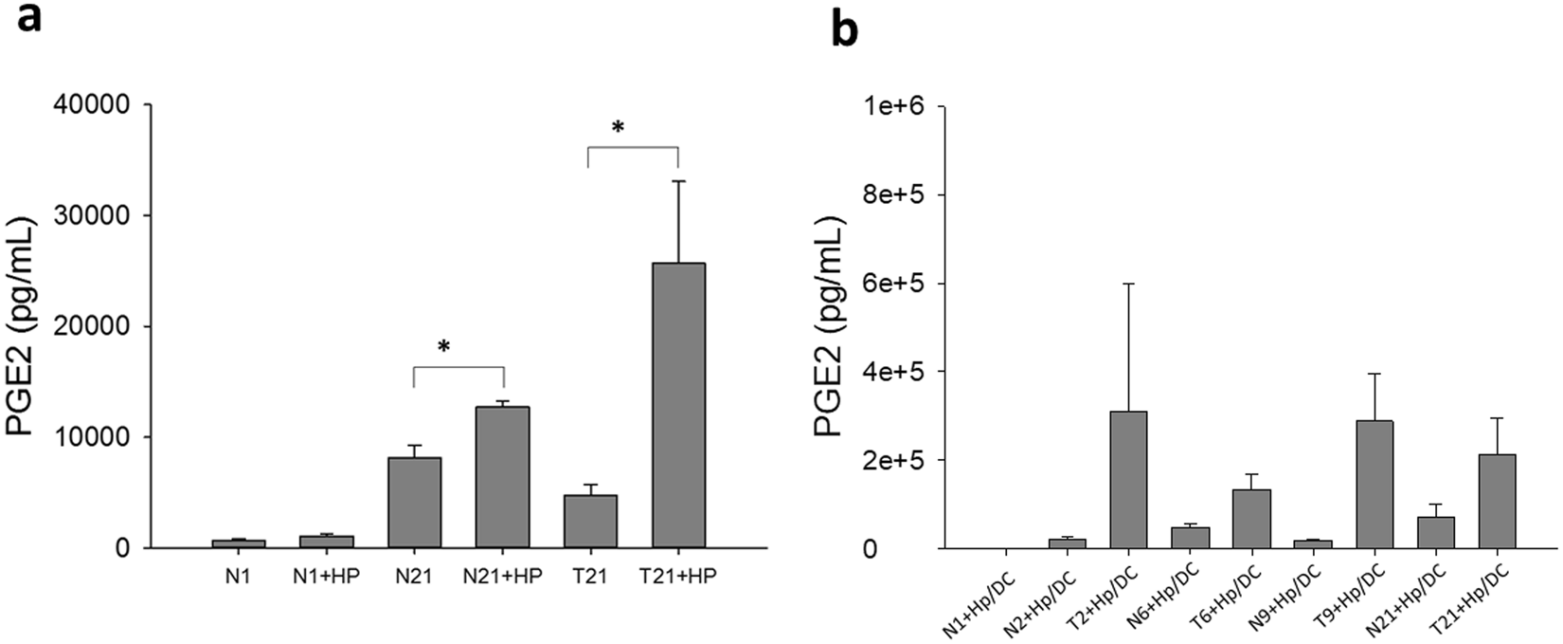
Soluble factor PGE_2_ secreted from Hp treated GSCs. (**a**) Plating 2 × 10^5^ GSCs cells/well with or without Hp infection (MOI = 1:200) for 24 hrs. (**b**) Using transwell co-culture experiment, plating 2 × 10^5^ GSCs cells/well with Hp infection (MOI = 1:200) in the apical side of transwell insert with a total of 2 × 10^5^ DCs were cultured in basolateral side of 24-well plates for 24 hrs. Supernatants were collected for PGE_2_ analysis by ELISA. GSCs N1 derived from a Hp negative, non-cancer control subject was used for reference. Paired GSCs of tumor (T2, T6, T9, and T21) and adjacent normal part (N2, N6, N9, N21) were obtained from gastric cancer patients. PGE_2_ data are expressed as mean ± SE of three-five independent experiments from different DC donors. Mann-Whitney U-test was used and for pair-wise comparisons only. *p < 0.05, **p < 0.01, ***p < 0.001.

**Figure 3:**
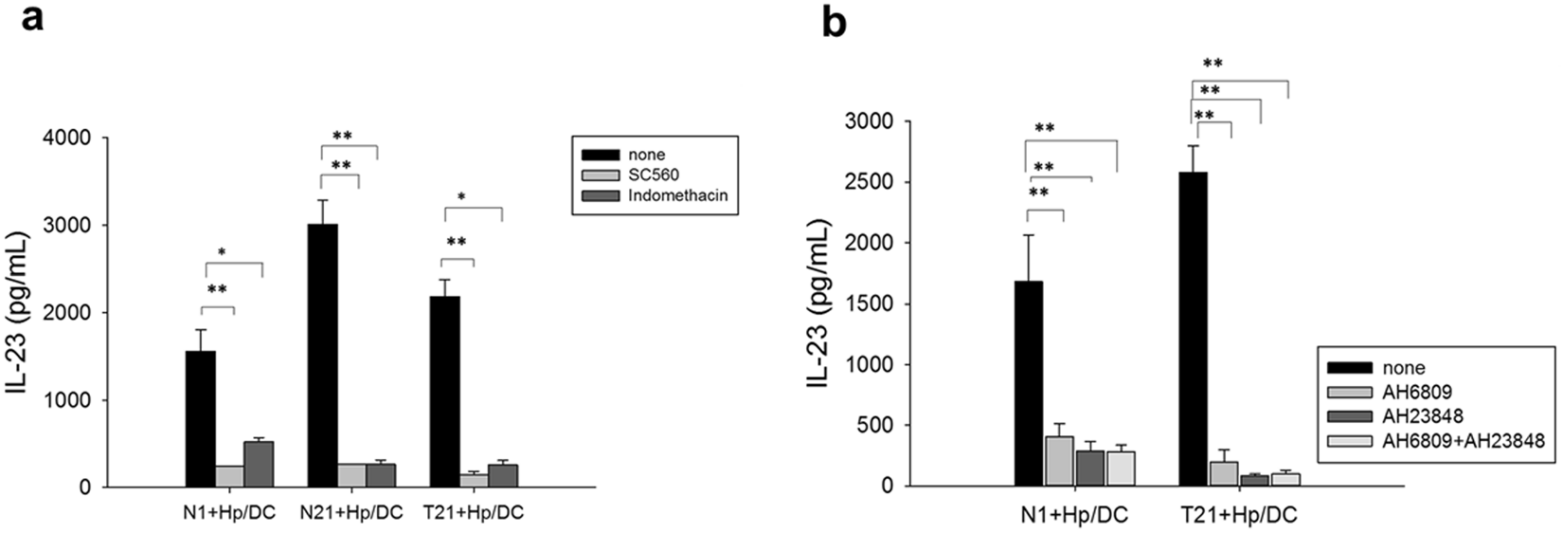
Effect of PGE_2_ in inducing IL-23 production in DCs. Transwell co-cultures were set up as above in the absence (none; vehicle control) or the presence of (**a**) COXs inhibitors (SC560, 100 nM, or indomethacin, 100 nM) or (**b**) PGE_2_ receptor antagonists (AH6809, 10 uM, and AH23848, 10 uM). Data given as the means ± SE from three-five independent experiments performed with DCs from different donors. Mann-Whitney U test was used and for pair-wise comparisons only. *p < 0.05, **p < 0.01.

**Figure 4:**
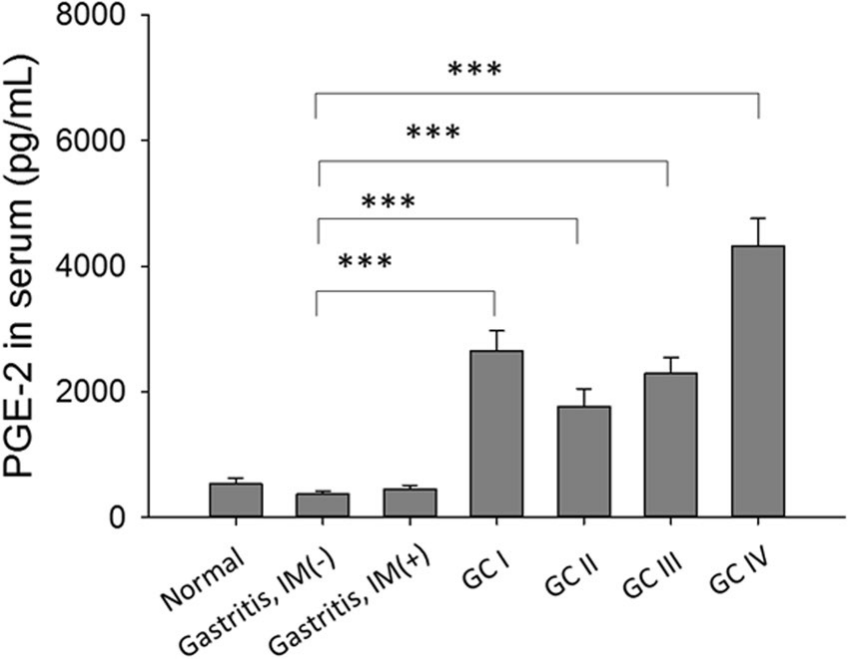
Levels of serum PGE_2_ in patients with gastritis without intestinal metaplasia [IM (−), n = 40)], gastritis plus intestinal metaplasia [IM (+), n = 34], gastric cancer (GC, n = 83) or healthy individuals (n = 10). Results are expressed as mean ± SE. Mann-Whitney U test was used and for pair-wise comparisons only. ***p < 0.001.

As PGE_2_, a lipid mediator, has been shown to play a key role in the regulation of inflammatory microenvironment in tumor tissues, we next examined whether PGE_2_ was generated from Hp-primed GSCs (Hp-GSCs) and could be one of those, thus far, a soluble factor capable of priming DCs. The same trans-well co-culture system described above was set up, and supernatants from the apical or basolateral side were collected for PGE_2_ measurement. When GSCs were stimulated directly with Hp, significantly increased PGE_2_ expression was found (Fig. 2a). Increased PGE_2_ expression in Hp-GSCs from cancer lesions (T2, T6, T9, T21) was also noted as compared with their respective adjacent normal tissues (N2, N6, N9, N21) of cancer patients (Fig. 2b). However, PGE_2_ expression from healthy individual GSCs N1 as a reference was negligible. In order to examine whether PGE_2_ was responsible for IL-23 induction in DCs, SC-560, a selective COX-1 inhibitor or a nonselective COX-1 and 2 inhibitor, indomethacin, was added to the culture. Results showed that IL-23 expression was inhibited in the presence of COX inhibitors, SC-560 and indomethacin (Fig. 3a), suggesting that PGE_2_ was critical in the induction of IL-23 expression in DCs. To further validate its effect, we applied PGE_2_ receptor antagonists, AH6809 (an EP2 receptor antagonist) and AH23848 (a selective antagonist for EP4), to block PGE_2_ signaling to determine whether PGE_2_-mediated IL-23 induction was via EP receptors on DCs. Our results indicated that the addition of either EP2 or EP4 antagonist could significantly abrogate the IL-23 production from the DCs (Fig. 3b). Interestingly, significantly increased levels of serum PGE_2_ were found (Fig. 4) in a panel of subjects with gastric cancer when compared to those with gastritis without intestinal metaplasia.

### COLEC12 expression in GSCs and gastric cancer tissues

**Figure 5:**
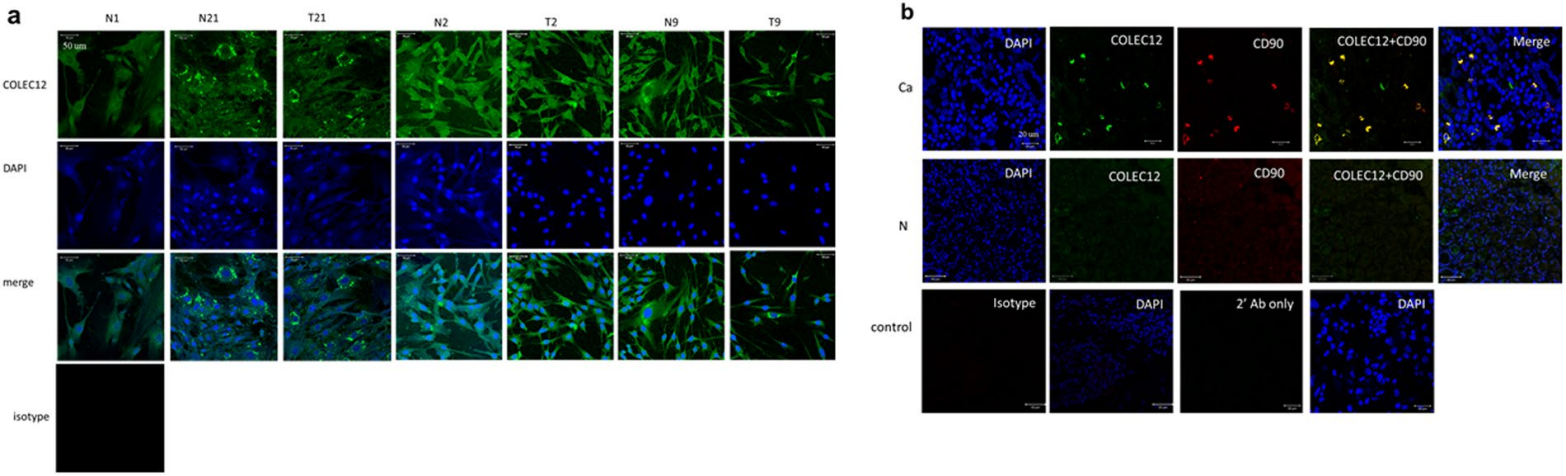
Immunofluorescence analysis of COLEC12 expression in (**a**) GSCs primary cell culture from various sources as indicated. GSCs were co-stained with rabit anti-COLEC12 primary Abs/Alexa 488 goat anti-rabbit secondary Abs (Green) and DAPI (Blue, for staining nucleus). An isotype control Ab was included as a negative control. (**b**) Immunofluorescence was used to analyze COLEC12 expression in gastric surgical tissue specimens from a gastric cancer patient T21 (Ca) and a non-cancer control individual N1 (N). COLEC12-positive cells are shown in green and those positive for CD90^+^ stromal cells are shown in red. Isotype control Abs and secondary Abs only were included as negative controls. GSCs N1 derived from an H. pylori-negative, non-cancer control subject was used for reference. The other three paired GSCs of tumor (T2, T9, and T21) and adjacent normal parts (N2, N9, N21) were from gastric cancer patients.

Next, we determined which innate pattern recognition receptor(s) expressed on GSCs was responsible for mediating Hp’s effect, focusing on a panel of CLRs according to our previous finding in GSCs gene expression microarray (data not shown). Results showed that GSCs expressed primarily COLEC12 and ORL1 among a panel of different CLRs, which were verified by Flow cytometry (supplemental Fig. 3a) and immunohistochemistry (supplemental Fig. 3b). By immunofluorescence, the expression of COLEC12 was clearly evident in GSCs with scattered expression pattern, in stromal cells or its adjacent normal GSC of subjects with gastric cancer (Fig. 5a). Further, immunofluorescence staining of COLEC12 and CD90^+^ stromal cells in gastric surgical tissue specimens demonstrated co-localization of COLEC12 in gastric stromal cells (Fig. 5b).

### COLEC12 on GSCs recognized H. pylori and primed DCs for IL-23 expression

**Figure 6:**
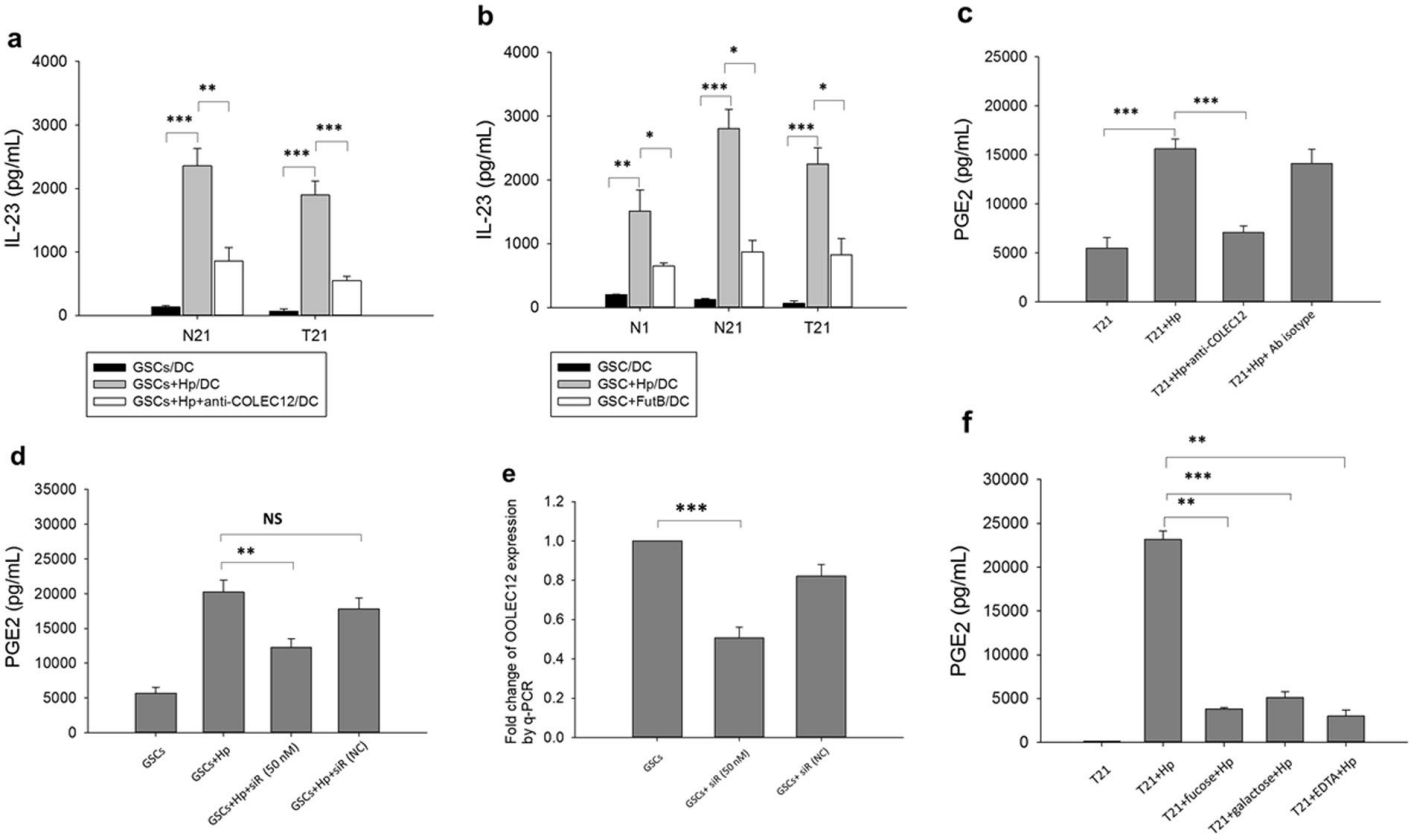
Recognition of Hp by COLEC12 in GSCs and its subsequent effect on DC-derived IL-23 expression. (**a**) Blockade of IL-23 expression in GSC-DC co-cultures by anti-COLEC12 blocking Abs (30 ug/ml). A trans-well co-culture system was set up as above in the absence or presence of anti-COLEC12 blocking Abs in the upper chamber, and the levels of IL-23 in the lower chamber was measured the same as in Fig. 1. (**b**) Levels of IL-23 secretion in the co-cultures with Hp wild-type strain (Hp) or a mutant strain of Hp lacking an α3-fucosyltransferase gene (FutB; HP0651). To evaluate the role of COLEC12 in GSCs in regulating PGE_2_ expression, GSCs were cultured with (**c**) anti-COLEC12 blocking Abs (30 ug/ml) for 1 hr or (**d**) COLEC12 siRNA (50 nM) for 72 hrs before Hp treatment. Twenty-four hrs after Hp stimulation, supernatants were collected and PGE_2_ expression was measured by ELISA. (**e**) As shown in Fig. 6d, GSCs cell pellet were collected and COLEC12 expression was assessed by q-PCR. (**f**) The glycan structure confers the recognition specificity of COLEC12 on GSCs. Blocking reagents, fucose (25 mM) or galactose (25 mM), were added in GSC cultures for 1 hr before Hp treatment. After 24 hrs, supernatants were collected for PGE_2_ measurement by ELISA. The results are expressed as mean ± SE, and the data represent three to five independent experiments from different DC donors. In Fig. 6d and e, data were from analyses of GSCs from three gastric cancer patients (T2, T9 and T21). Mann-Whitney U-test was used and for pair-wise comparisons only. ANOVA followed Scheffe test of multiple post hoc analyses was used for q-PCR experiment. *p < 0.05, **p < 0.01, ***p < 0.001, NS; non significant.

To examine the role of COLEC12 in mediating Hp’s effect on GSCs and the secretion of IL-23 from DCs, similar Hp-GSC/DC co-cultures as described above with anti-COLEC12 blocking Abs in apical side of the trans-well were used. Results showed that the levels of IL-23 in the co-cultures of Hp-GSCs and DCs were significantly lower in the presence of anti-COLEC12 Abs than those without the addition of anti-COLEC12 Abs (Fig. 6a). In addition, when an alpha3-fucosyltransferase-null (*futB*; HP0651) strain of Hp was used to stimulate GSCs, the IL-23 expression in DCs was significantly decreased (Fig. 6b). Significantly downregulated PGE_2_ expression from GSCs was also found when anti-COLEC12-blocking Abs were added (Fig. 6c) or when GSCs with COLEC12 knockdown (Fig. 6d) were used. The efficiency of COLEC12 knockdown was assessed in GSCs from three different individuals by q-PCR (Fig. 6e). Moreover, the enhanced level of PGE_2_ was inhibited by the addition of a calcium chelator, EDTA, and was significantly reversed by the addition of fucose or galactose (Fig. 6f). These results suggested, therefore, the importance of COLEC12 as a pattern recognition receptor for Hp through their LPS’ glycan structures.

## Discussion

In this study, we found that a member of the CLR family, COLEC12, may play a role in Hp-mediated crosstalk between GSCs and DCs. It shares with another member of the CLR family, DC-SIGN, the ability to bind the Le^x^ epitope, but it interacts primarily with the galactose moiety of the glycan rather than the fucose residue^32^. Also, COLEC12 shares with the asialoglycoprotein receptor the ability to mediate endocytosis and degradation of glycoprotein ligands. While the functional significance of the COLEC12-lignad axis remains to be elucidated, our current data suggest its likely importance in the recognition of Hp by GSCs and its functional impact on GSCs and DCs, which may represent a critical regulatory mechanism in Hp-mediated immune response.

In this study, we evaluated the viability of gastric stromal cells in presence of varying doses of Hp at 24-hr time point by MTT assay, and found that at a higher dose (200 MOI), the viability was 83%-99% (supplemental Fig. 4a). Further, we found that lower doses of Hp (MOI = 50–100) induced, in fact, higher levels of PGE_2_ from GSCs than those of the cells treated with a higher dose of Hp (200) (supplemental Fig. 4b). The lower levels of PGE_2_ release could be due to the higher level of cytotoxicity. It is noted that in addition to GSC-derived soluble factors, Hp-derived outer membrane vesicles (OMVs) in the co-cultures could play a role in stimulating DCs as it has been reported that the size of OMVs is ranged from 20 to 300 nm, significantly smaller than the transwell insert (0.45 um)^33,34^. In our trans-well co-cultures, the amount of OMVs in the Hp (MOI = 1:200) culture medium was calculated to be 10 ug based on their protein content. The increased levels of PGE_2_ secreted from GSCs were noted only when GSCs were stimulated with over 200 ug of OMVs (supplemental Fig. 4c), suggesting that OMVs at the dosage below 200 ug might not be critical, although a potential additive effect of very low doses of OMVs and Hp cannot be ruled out at this time. Moreover, in our co-culture experiments, the induction of DC-derived IL-23 could be blocked by PGE_2_ receptor antagonists or in trans-well culture conditions where GSCs with COLEC12 knockdown or the addition of anti-COLEC12 Abs. These results supported, therefore, the contention that the increase of IL-23 in DCs could be, at least in part, mediated through a priming event originated from Hp-infected GSCs.

Lewis x and Lewis y antigens are the main antigens expressed in the LPS of Hp strains^35^, which are known to display phase variation by reversible on-off switching of genes controlling LPS biosynthesis^36^. The α3 fucosyltransferase is associated with phase variation of LPS, and there are two α3 fucosyltransferase genes (*futA* and *futB*) in Hp 26695, and α3 fucosyltransferase knockout experiment has revealed that only Hp stains with intact *futB* reading frame can express Lewis x and Lewis y^37^. In this study, when compared with wild-type strain 26695, GSCs infected with *futB* knockout strain showed significantly reduced capacity in priming DCs for secreting IL-23. Further, Hp-induced PGE_2_ in GSCs could be partially inhibited by the addition of COLEC12-blocking Abs or in GSCs with COLEC12 knockdown. While in our positive control experiments, successful transfection (supplemental Fig. 5) and potent GAPDH RNAi inhibitory activity were found (data not shown). The results from these sets of control experiments suggested that the siRNA sequence for COLEC12 currently used were suboptimal. Alternatively, the partial inhibitory effect of Ab blockade or gene knockdown may suggest the existence of additional, yet unidentified, receptors on GSCs, such as TLRs, for recognition of Hp, which awaits further investigation. Nevertheless, these results, collectively, support the potential importance of COLEC12 in conferring GSC’s recognition of Hp Lewis antigen and its subsequent impact on DC’s functions. It was also found that increased cytosolic levels of COLEC12 expression were seen in cancer-associated stromal cells, but the reason for this is, at present, unclear. It could be that the cytosolic COLEC12 might represent those that have been internalized upon binding to fucosylated endogenous ligands, as a part of its “clearance” mechanism for removing fucosylated ligands^32^. This could be due, in part, to an increased fucosyltransferase activity in the stromal cells or cancer cells from patients, and hence an increased level of COLEC12 ligands.

Accumulating evidence has indicated that the inflammatory responses are important for cancer development, wherein COX-2 and its downstream product, PGE_2_, are suggested to play a key role in gastrointestinal tumorigenesis, particularly in mouse gastric tumor models^38,39^. Furthermore, activation of COX-2 ⁄PGE_2_ pathway in gastric cancer cells together with TLR⁄MyD88 signaling in DCs have been shown to be a part of the inflammatory microenvironment^38,39^. Our study demonstrated that PGE_2_ was the important soluble factor released from Hp-infected gastric stromal cells, in a COLEC12-dependent manner, and was responsible in conditioning DCs and generating subsequent Th17 response. While the exact mechanism for the enhanced levels of PGE_2_ in cancer-associated GSCs was, at present, unclear, these findings provide evidence supporting the existence of a potentially critical stromal cell-associated inflammatory microenvironment in perpetuating the tumorigenesis.

It was also noted that GM-CSF secreted from Hp-GSCs was found to be important in regulating PGE_2_ levels. Interestingly, in the presence of anti-GM-CSF neutralizing Abs, the levels of PGE_2_ were significantly higher in comparison to those without the addition of anti-GM-CSF Abs (Supplemental Fig. 6). As a corollary, GM-CSF was found to be able to reduce PGE_2_ secretion from stromal cells of the bovine endometrium^40^ and also from GM-CSF-primed bone marrow-derived macrophages stimulated with TLR2 ligand^41^. Therefore, it appears that GM-CSF can negatively regulate PGE_2_ expression.

Increasing evidence has revealed the importance of IL-23 in linking innate and adaptive immunity, possessing important roles in the differentiation and initiation of both Th1 and Th17 responses^42^. Similar to our results, other studies have indicated that PGE_2_ enhances the production of IL-23^30,43–45^. Interestingly, Bimczok’s study^10^ indicated that gastric stromal factors, independent of PGE_2_, down-regulate DC responsiveness to H. pylori and dampen gastric Th1 response, contributing to the permissiveness of persistent residential microbes. Our study, in fact, suggested that GSC-derived PGE_2_ is critical in eliciting DC’s IL-23 expression and the subsequent Th17 response.

## Conclusion

**Figure 7:**
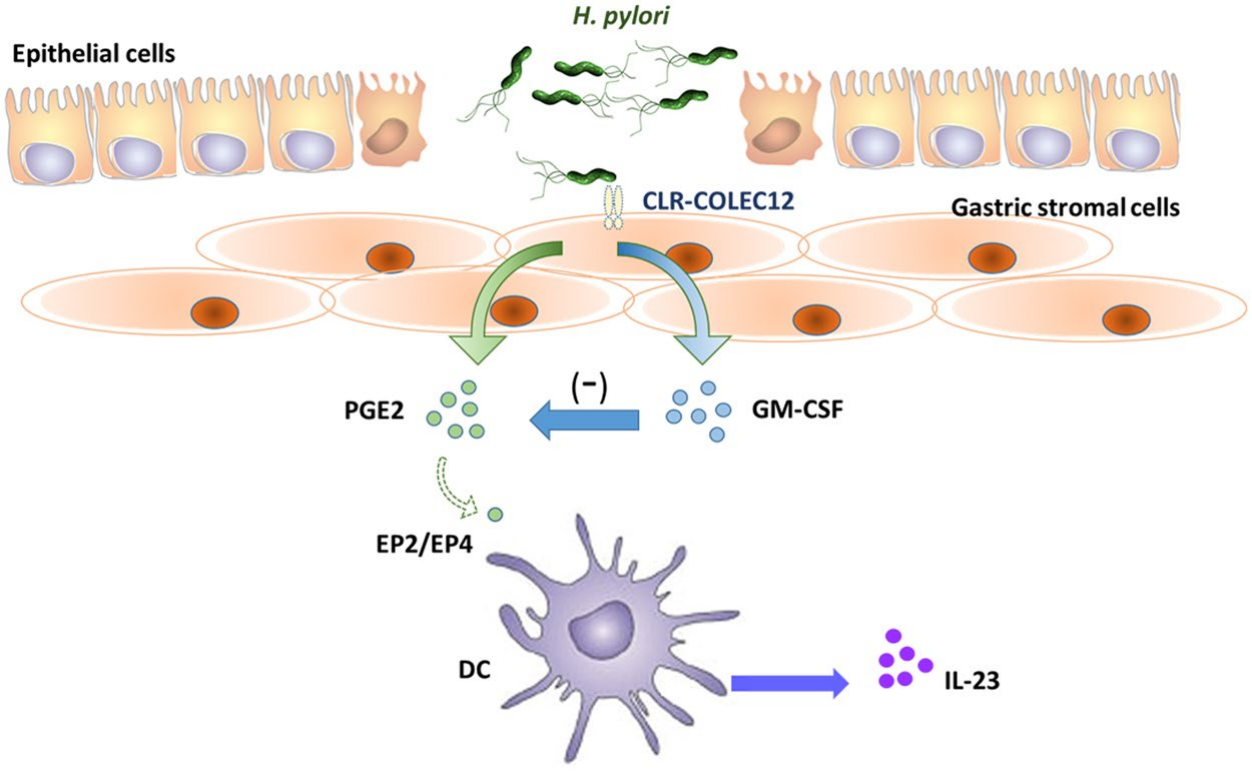
A schematic diagram illustrates a novel regulatory pathway controlling gastric mucosal immunity, wherein a C-type lectin receptor (CLR), COLEC12, is important in bridging *H. pylori*-mediated crosstalk between gastric stromal cells (GSCs) and DCs within the gastric mucosa. Hp can disrupt the epithelial barrier and transit to the sub-epithelial mucosal layer, where the contact between Hp and GSCs is possible and critical in controlling Hp-mediated inflammation. Subsequent to the Hp-stromal cell interaction, PGE_2_-dependent conditioning effect on DCs is noted and PGE_2_ released from Hp treated GSCs is negatively regulated by GM-CSF and shown to be able to induce IL-23 secretion in DCs.

This study identified a significant cross-talk between GSCs and DCs, in which PGE_2_ released from Hp treated GSCs was shown to be able to induce IL-23 secretion in DCs and modulate IL-17 expression in T cells. A schematic diagram summarizing this new regulatory pathway is presented in Fig. 7. Interestingly, COLEC12 on GSCs appeared to be responsible, in part, in mediating the effect of Hp on GSCs. Further, the COLEC12-mediated impact was decreased when the cells were stimulated with a mutant strain of Hp lacking α3 fucosyltransferase gene (*futB)*, suggesting that the glycan structures decorated with fucose/galactose significantly confer the recognition specificity of COLEC12 on GSCs. Taken together, these results provide a strong case supporting the likely importance of COLEC12 as a receptor involved in Hp-stromal cell interaction and in its subsequent conditioning and programming effect on DCs, where the LPS Le glycoform variants play a key role.

## Electronic supplementary material

supplementary data
