# Supplementary material for: Stromal C-type lectin receptor COLEC12 integrates H. pylori, PGE2-EP2/4 axis and innate immunity in gastric diseases
Source: Sci Rep. 2018 Feb 28;8:3821. doi: 10.1038/s41598-018-20957-2 (PMC5830506; doi:10.1038/s41598-018-20957-2)
Supplement: Supplementary file 1 — supplementary data [file 41598_2018_20957_MOESM1_ESM.pdf]

Stromal C-type lectin receptor COLEC12 integrates H. pylori, PGE2-EP2/4 axis and innate immunity in gastric diseases

Lin-Li Chang, PhD [1-5], Wen-Hung Hsu, MD[2,6], Mou-Chieh Kao, PhD[7,8], Chih-Chung Chou[1], Chung-Cheng Lin[2], Chung-Jung Liu, PhD[6], Bi-Chuang Weng[6], Fu-Chen Kuo, MD, PhD[9], Chao-Hung Kuo, MD, PhD[2,6,10], Ming-Hong Lin[1], Chun-Jen Wang [7], Chun-Hung Lin, PhD[11-13], Deng-Chyang Wu, MD, PhD[2,4,6,10,14*], Shau-Ku Huang, PhD[15, 16,17,18*]

Supplemental Figure 1

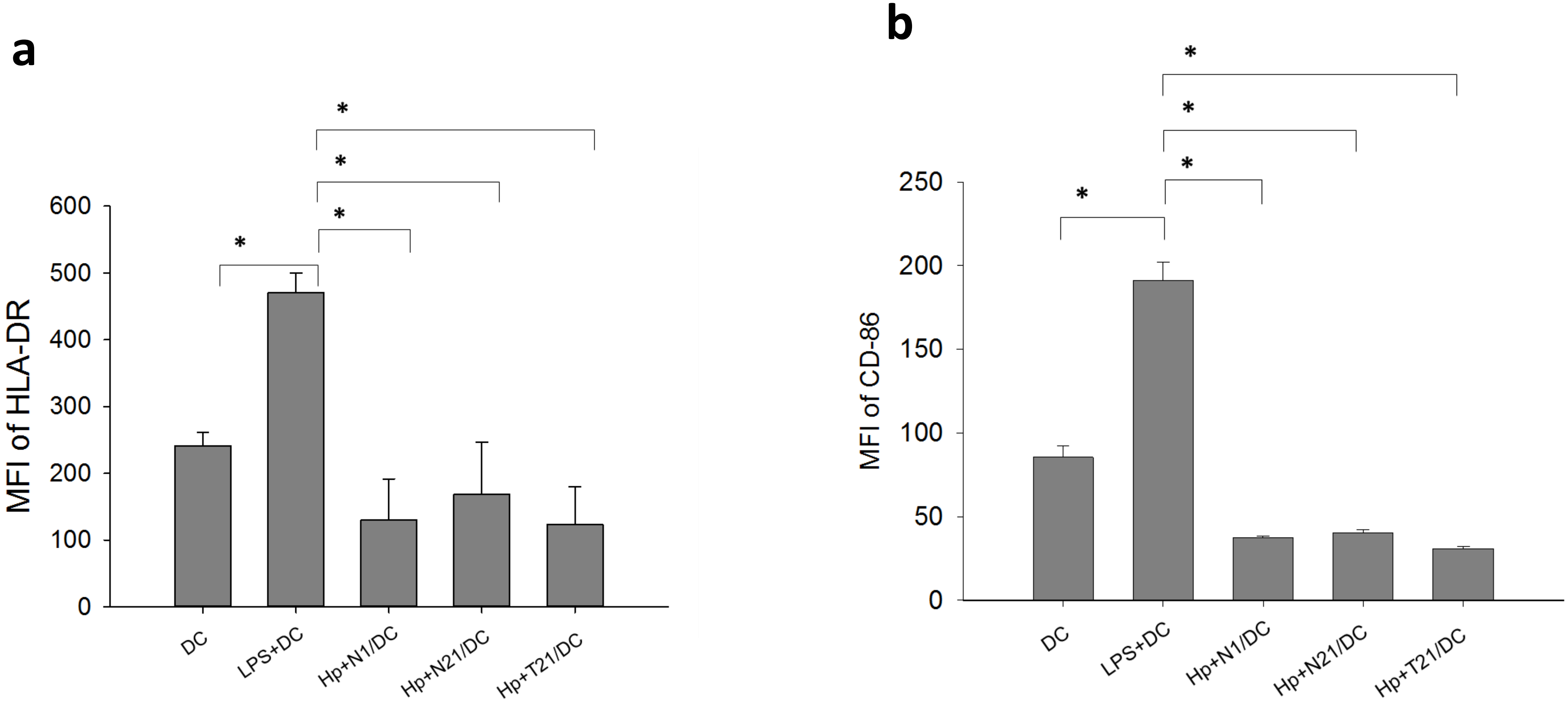

Supplemental Figure 1. Expression of surface markers on DCs co-cultured with H. pylori-primed GSCs from a gastric cancer patient, T21, its adjacent normal gastric stromal tissue N21, and a healthy individual, N1, for 24 hrs. Mean fluorescence intensity (MFI) expression of (a) HLA-DR, (b) CD86 in DCs was detected by flow cytometry. The results are expressed as means ± SE, and the data shown represent independent experiments from three-five different DC donors. Mann-Whitney U test was used. *p<0.05 in pair-wise comparisons as indicated by bracket symbols

Supplemental Figure 2ab

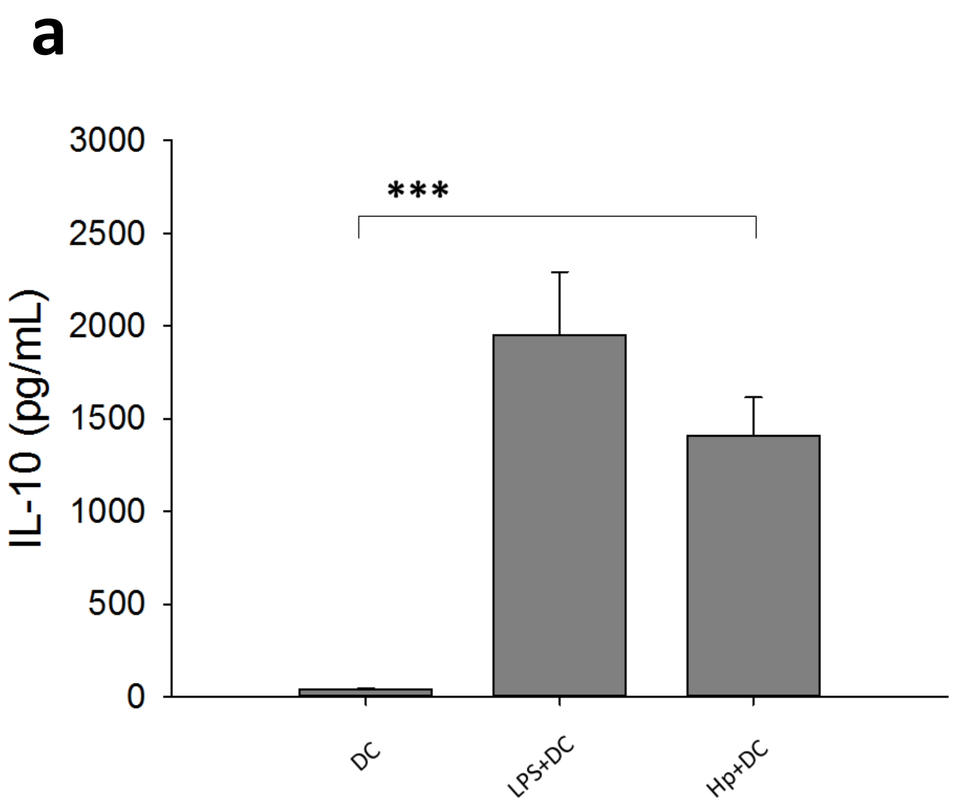

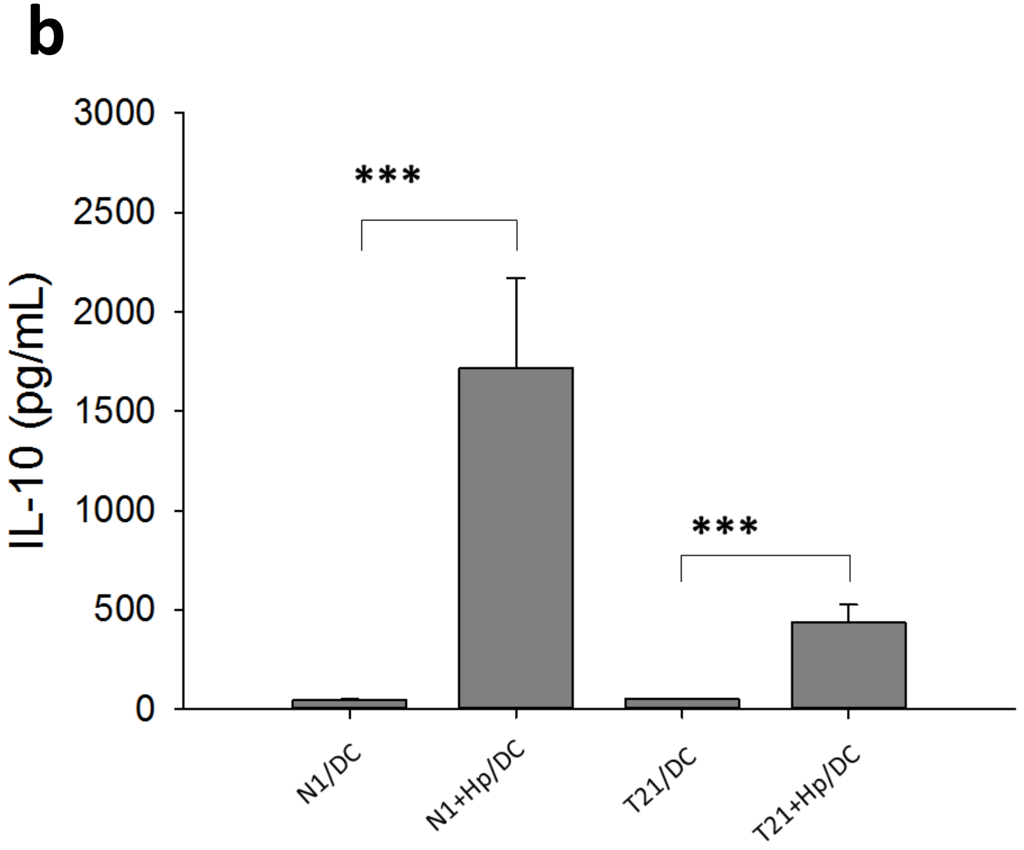

Supplemental Figure 2cd

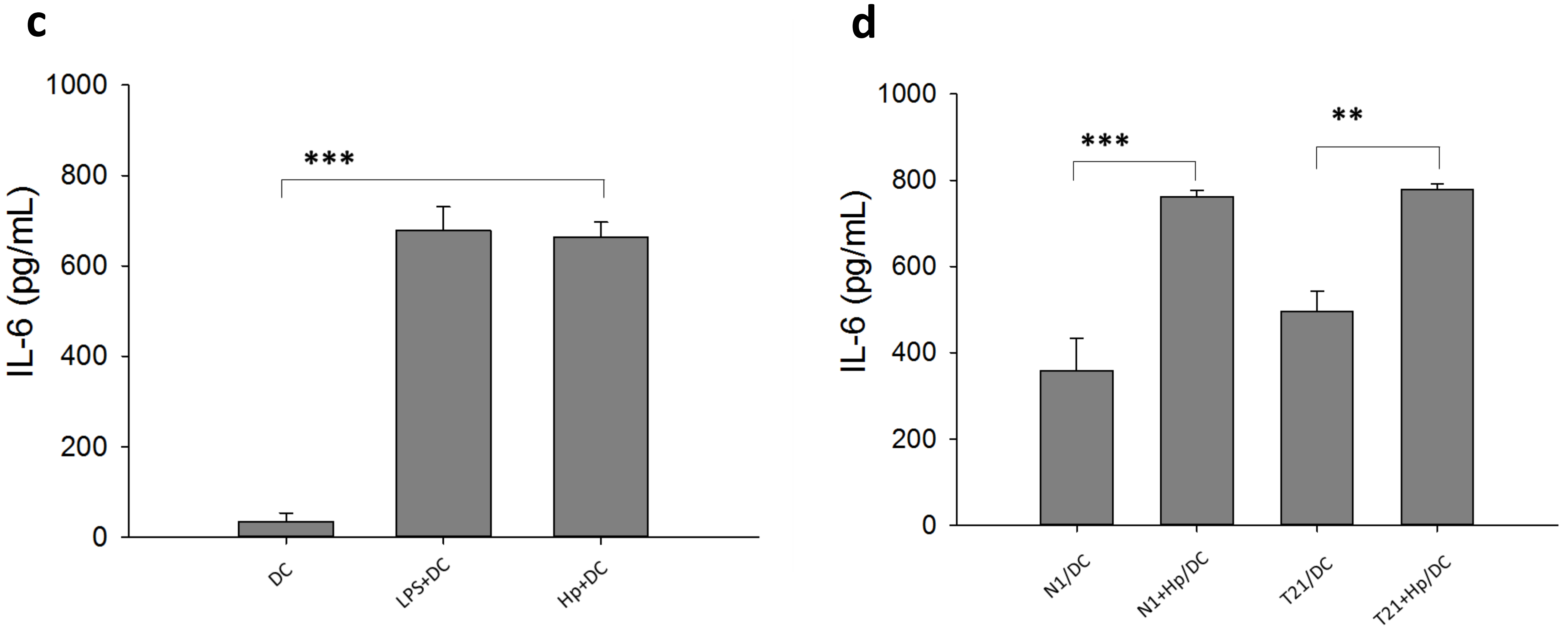

Supplemental Figure 2ef

e

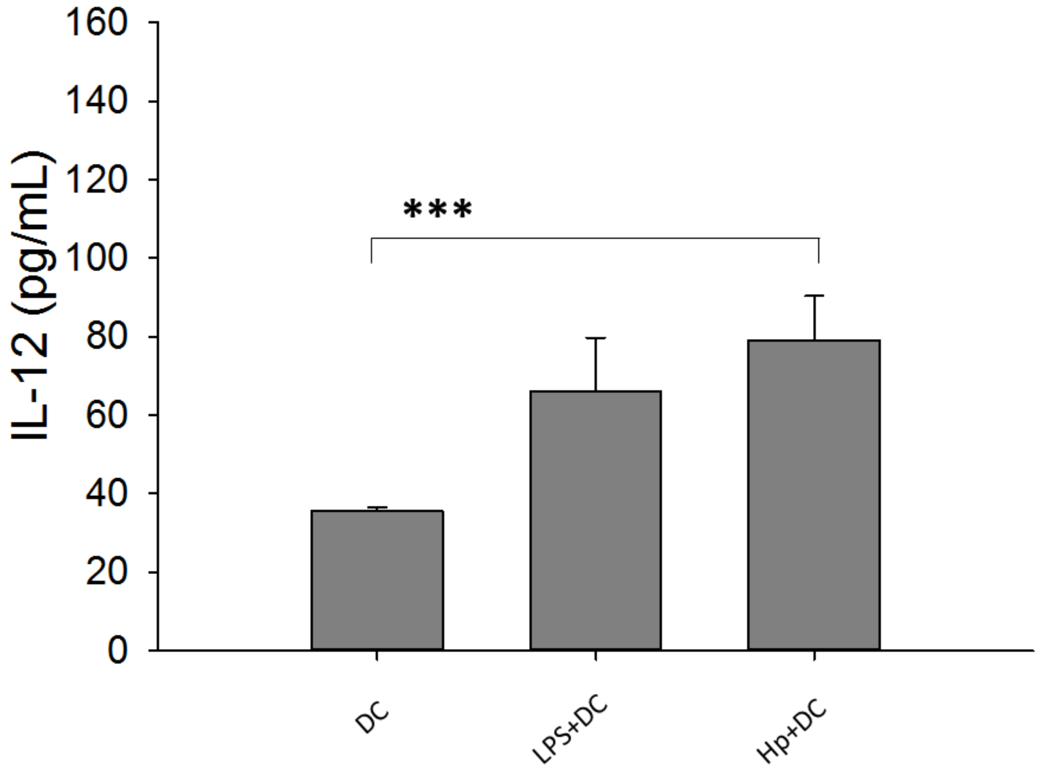

f

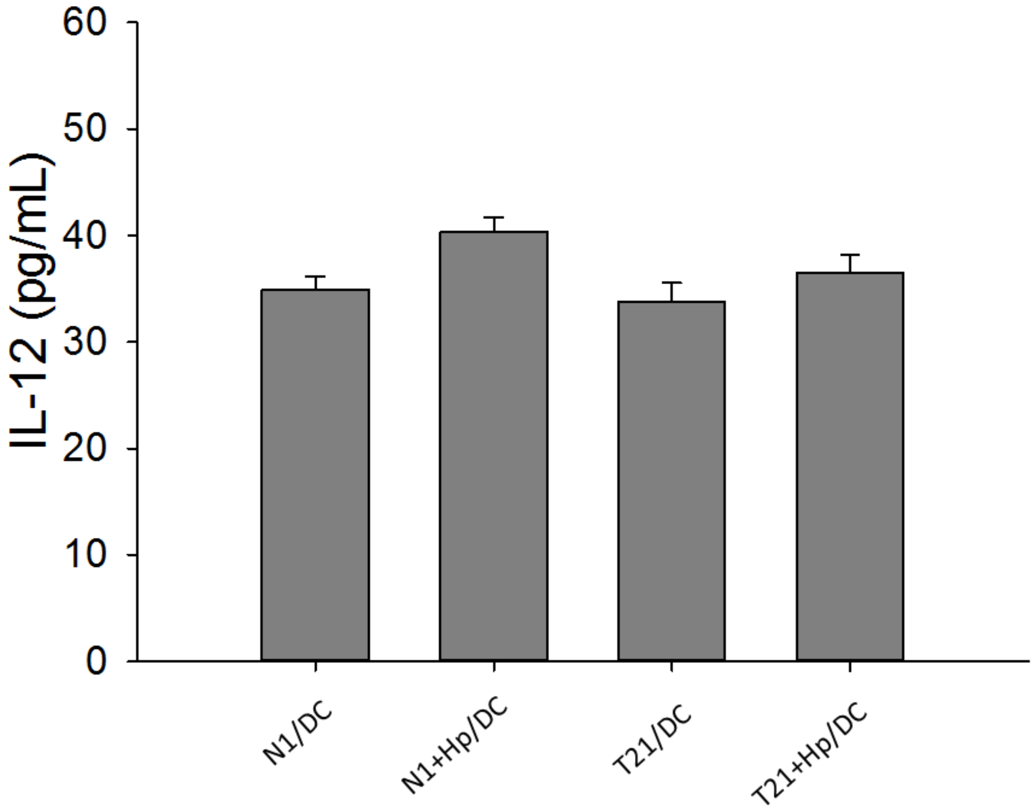

Supplemental Figure 2gh

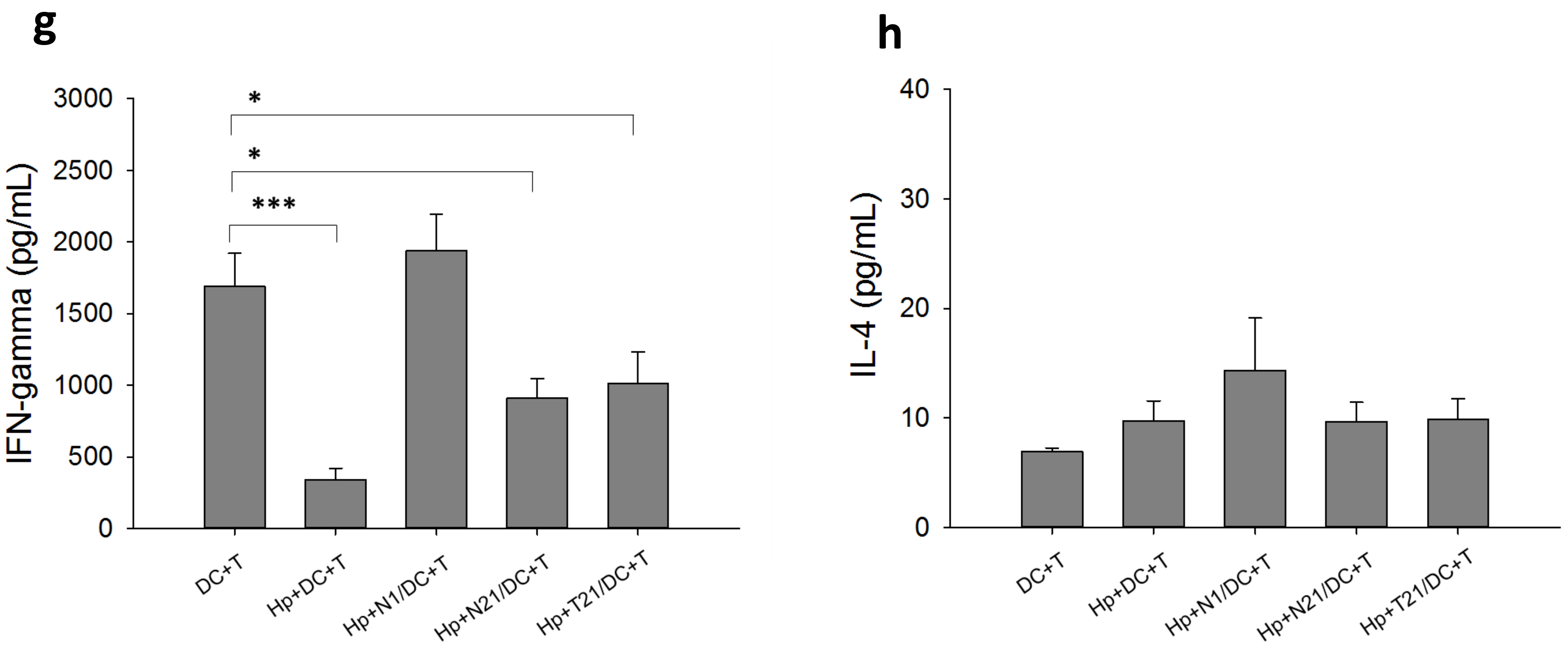

Supplemental Figure 2. Cytokines secretion from DCs in trans-well co-cultures with GSCs. The level of (a and b) IL-10, (c and d) IL-6, and (e and f) IL-12 expression from DCs in trans-well co-cultures with GSCs of a non-cancer control individual (N1) and a cancer patient (T21, GSCs from cancer lesion) in the absence or presence of Hp stimulation (Hp:MOI=1:200) was analyzed by ELISA. Hp were added for 24 hrs either in the upper chamber for stimulation of GSCs ($2x10^5$ cells/ml) or in DC ($2x10^5$ cells/ml) culture alone for activation of DCs directly. E. coli-derived LPS (100 ng/ml) was used for comparison. Hp-GSCs-conditioned DCs regulated IFN-γ and IL-4 expression from $CD4^+$ T cells was measured. Levels of (g) IFN-γ, (h) IL-4 expression in $CD4^+$ T cells co-cultured with conditioned DCs at a 1:5 ($DC/CD4^+$ T) ratio. The results are expressed as means ± SE, and the data shown represent independent experiments from three-five different DC donors. Mann-Whitney U test was used. *$p<0.05$, **$p<0.01$, ***$p<0.001$ in pair-wise comparisons as indicated by bracket symbols.

Supplemental Figure 3a

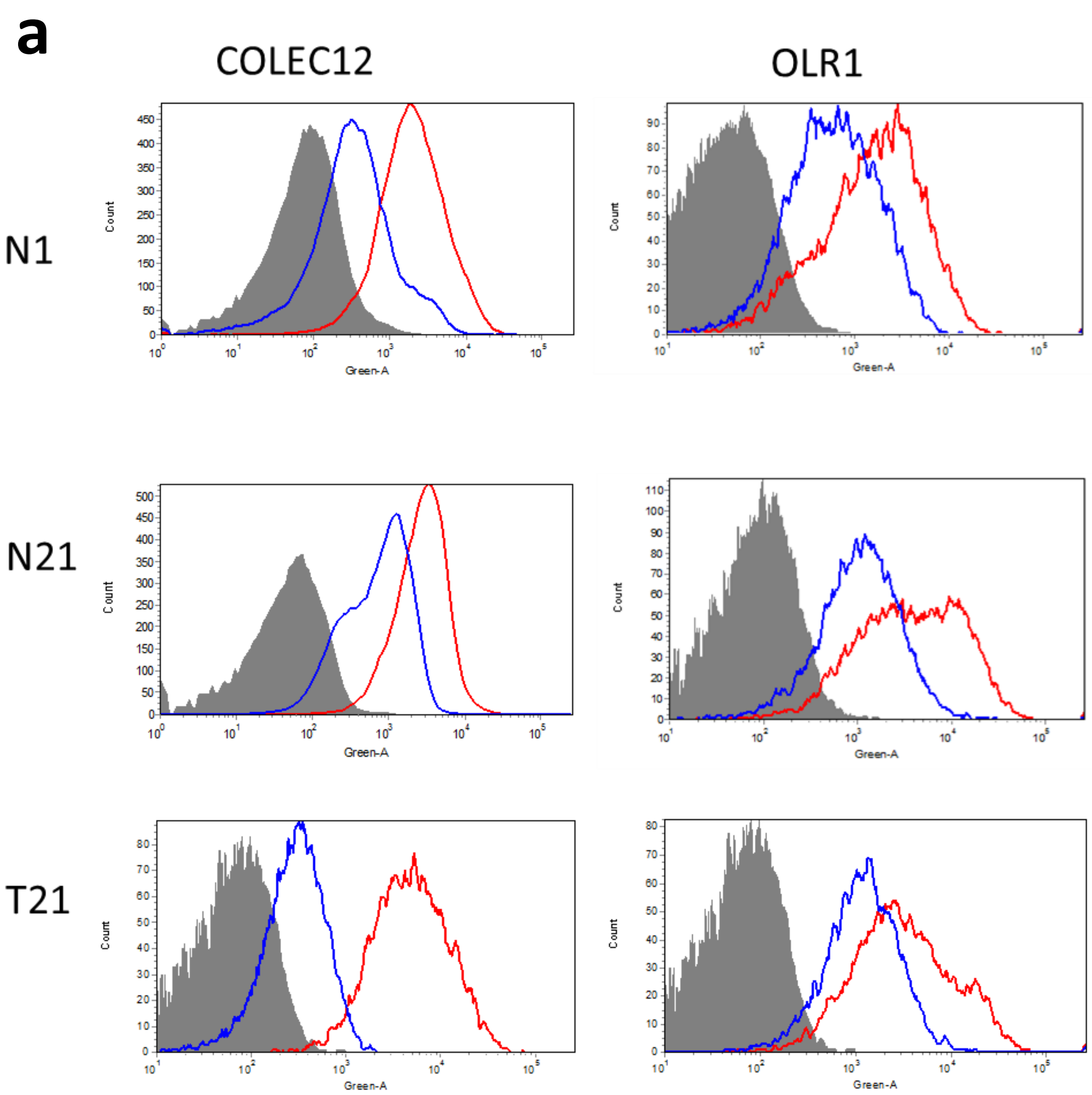

Supplemental Figure 3b

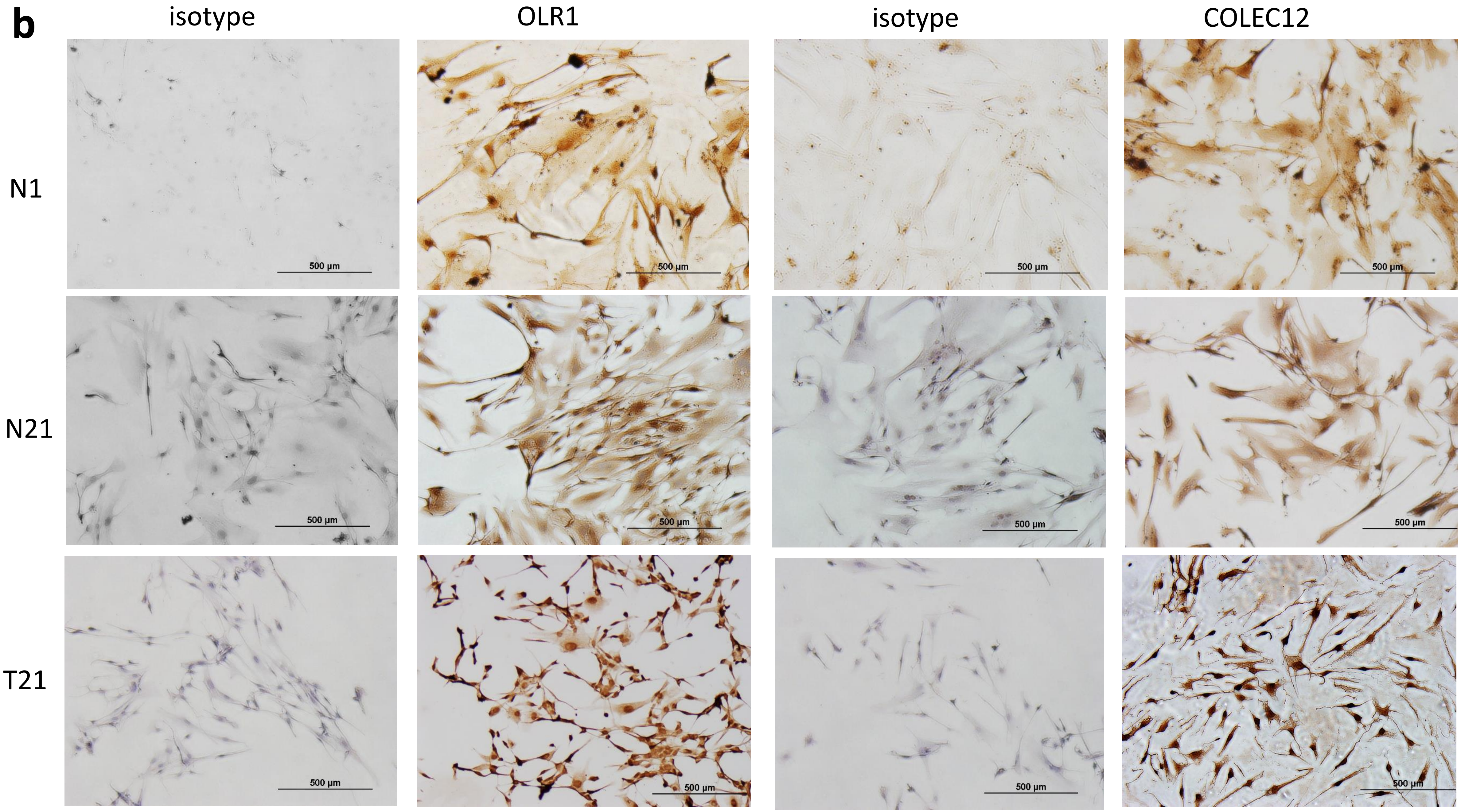

Supplemental Figure 3. Expression of COLEC12 and OLR1 on GSCs by (a) flow-cytometry and (b) immunohistochemistry. For flow-cytometry, $1x10^5$/ml GSCs were incubated with mouse anti-COLEC12 or mouse anti-OLR1 Abs for 30 minutes. Secondary Abs, phycoerythrin conjugated goat anti-mouse IgG, were added for 30 minutes. For immunohistochemistry, $1x10^5$/ml GSCs cultured on slides were incubated with the primary Abs described above for 24 hrs at 4°C. GSC N1 were from a non-cancer control individual, GSC T21 were from cancer lesion and GSC N21 from normal adjacent gastric cancer tissue. In panel A, the cells were incubated with the respective primary Abs (red line), isotype control (blue line) or medium control (gray shadow).

Supplemental Figure 4ab

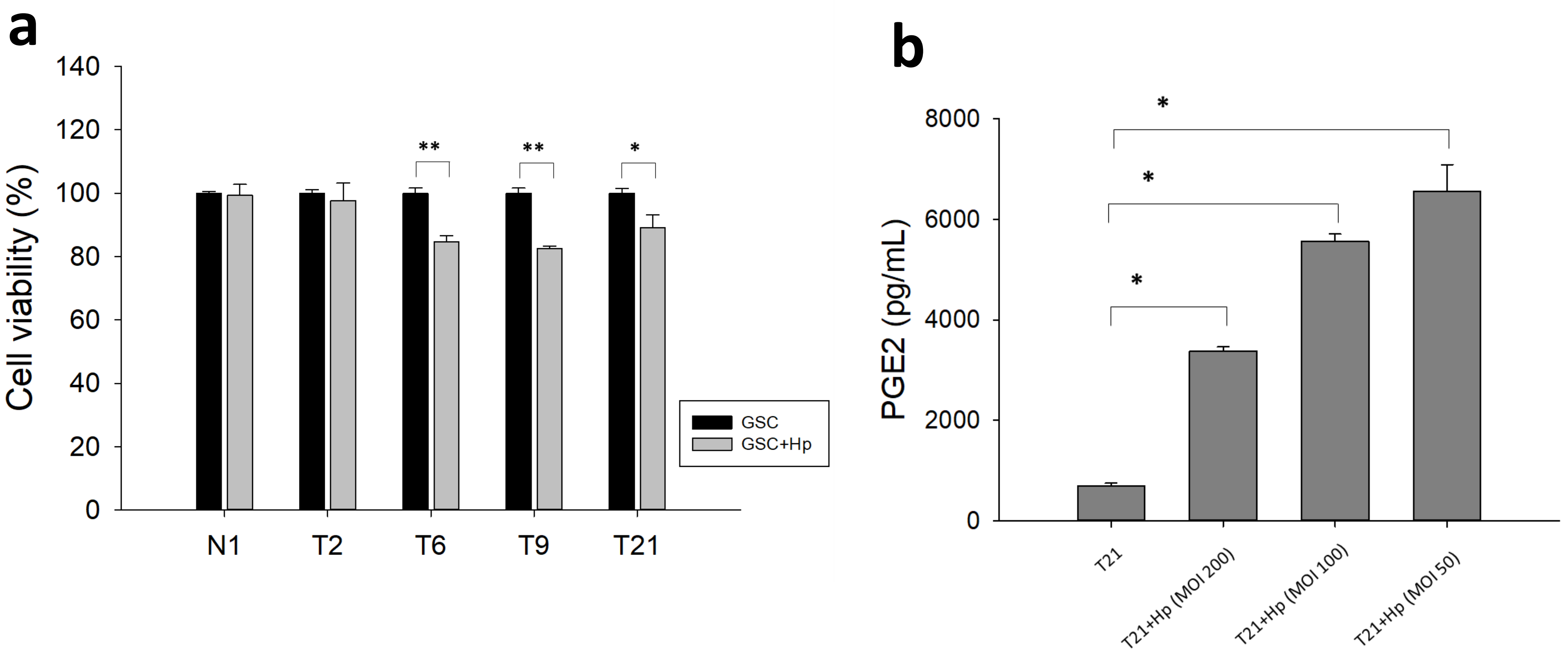

Supplemental Figure 4c

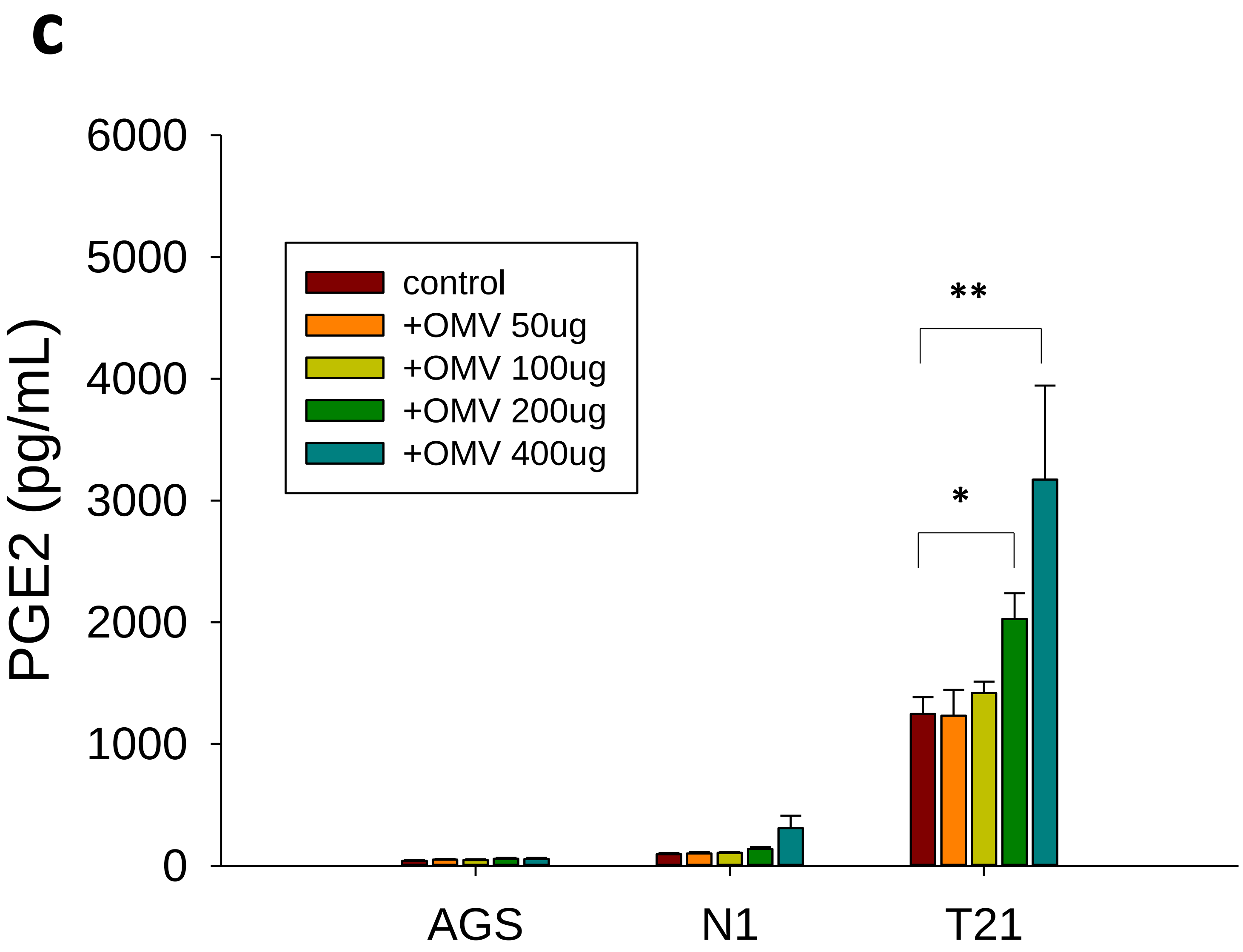

Supplemental Figure 4. Analysis of cell viability and PGE_2 secretion. (a) Analysis of cell viability by MTT assay of gastric stromal cells (GSCs) from gastric cancerous (GSC T21), their adjacent normal tissues (GSC N21) and a non-cancer patient (GSC N1) when cultured with Hp (MOI=200) at 24-hr. The results are expressed as means ± SE. Mann-Whitney U test was used. *p<0.05, ***p<0.001 vs the respective GSC alone as indicated. (b) PGE_2 secretion from Hp-primed GSC T21 (MOI=50, 100 and 200) was measured by ELISA assay. The results are expressed as means ± SE. Mann-Whitney U test was used. *p<0.05 vs the cell (T21) alone without stimulation. (c) PGE_2 released from Hp OMV-treated gastric epithelial cell (AGS) or gastric stromal cells (GSCs, N1, T21). Cells were grown alone or primed with different doses of Hp OMV (50 ug- 400 ug) for 24 hrs. Supernatants were collected for analyses of PGE_2 secretion by ELISA. The results are expressed as means ± SE. Mann-Whitney U test was used. *p<0.05, **p<0.01 vs control without the addition of OMVs.

Supplemental Figure 5

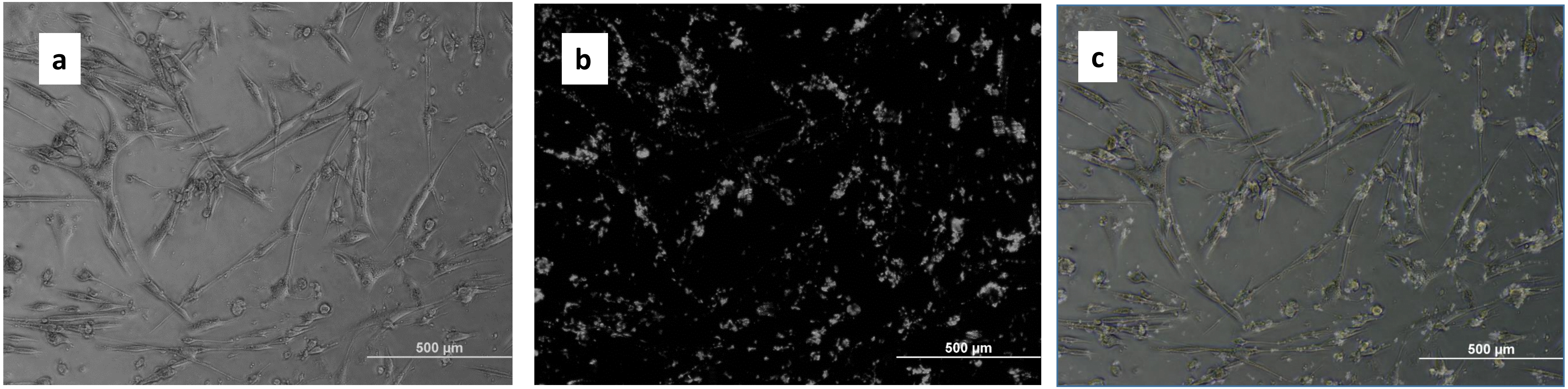

Supplemental Figure 5 SiGLO Green fluorescent oligonucleotide (Dharmacon) transfection indicators was successfully transfected into primary gastric stromal cells T21 (GSC T21) using TransIT-X2® Dynamic Delivery System (Mirus). The above images were taken 24 hours post-transfection and show high transfection efficacy in cells. (a) bright field microscopy image, (b) fluorescence image, (c) merged image.

Supplemental Figure 6

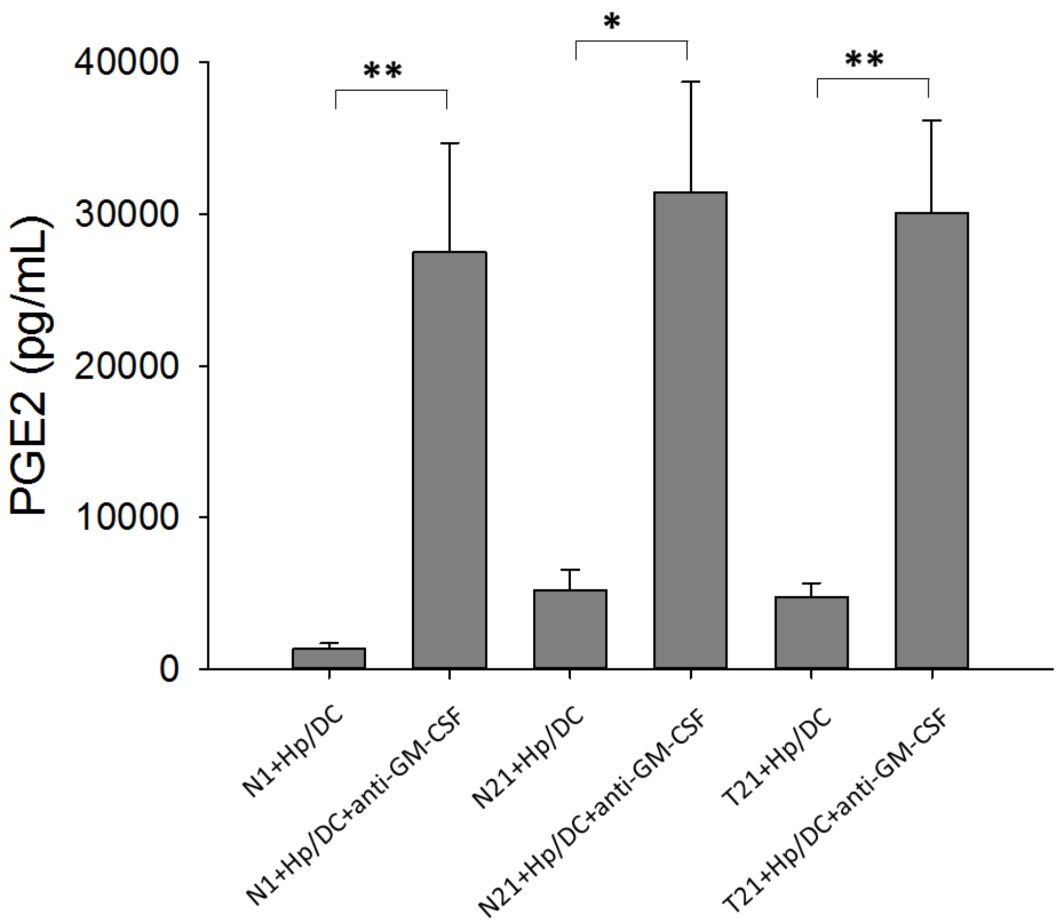

Supplemental Figure 6 Using transwell co-culture experiment, in the absence or presence of blocking GM-CSF antibodies, for 24 hrs. Supernatants were collected for PGE_2 analysis by ELISA. Data are mean±SE of three-five independent experiments. The differences between groups were compared by ANOVA. *p<0.05, **p<0.05.
